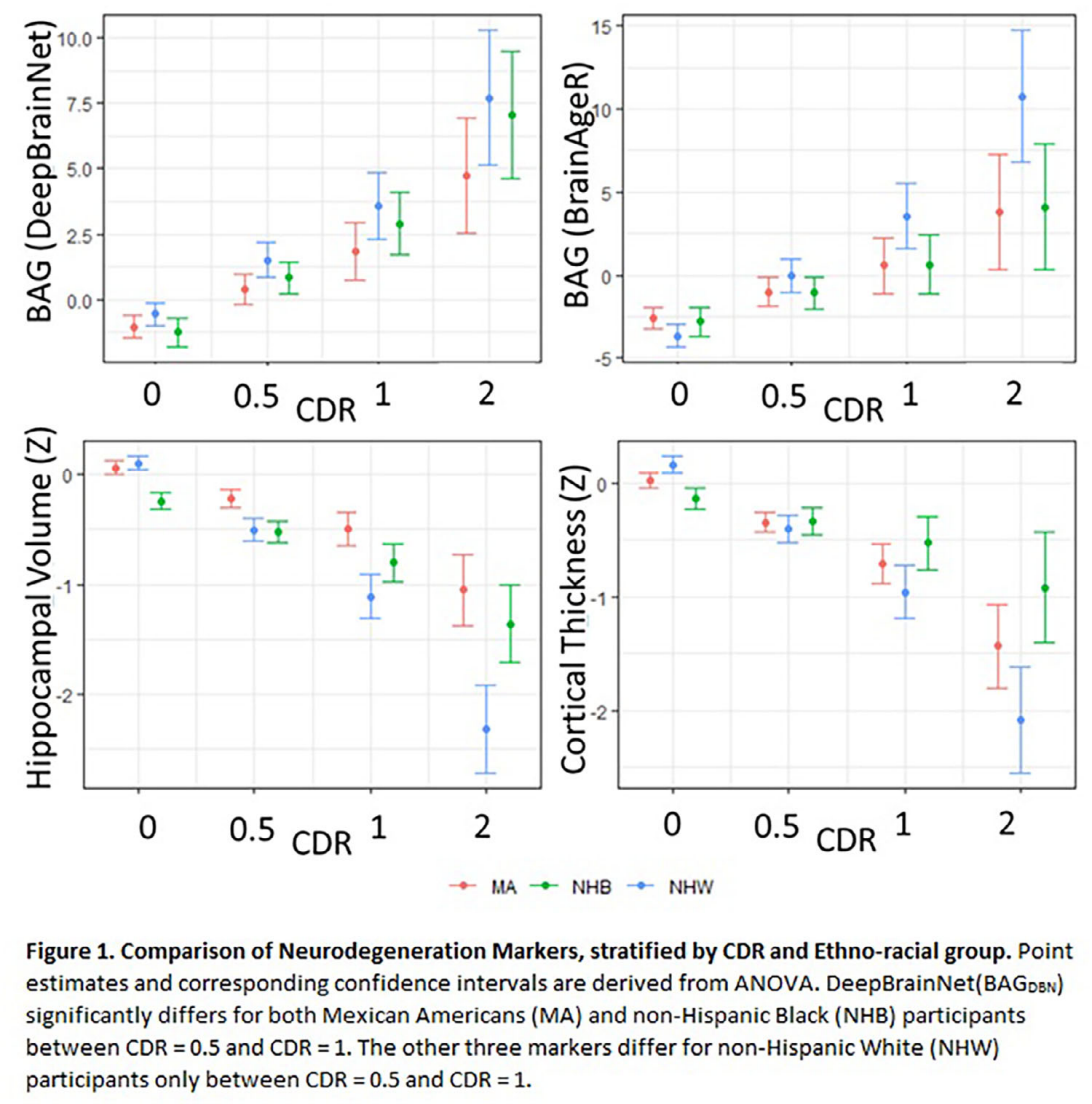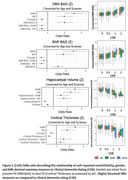# Cross‐Sectional Comparison of Structural MRI Markers of Cognitive Impairment in an Ethno‐racially Diverse Cohort

**DOI:** 10.1002/alz.086629

**Published:** 2025-01-09

**Authors:** Julie K. Wisch, Kalen Petersen, Peter R Millar, Omar Abdelmoity, Karin L. Meeker, Meredith N Braskie, Arthur W. Toga, Sid E. O'Bryant, Beau Ances, the HABS‐HD Study Team

**Affiliations:** ^1^ Washington University in St. Louis School of Medicine, St. Louis, MO USA; ^2^ Washington University School of Medicine, St. Louis, MO USA; ^3^ Washington University, St. Louis, MO USA; ^4^ Stevens Neuroimaging and Informatics Institute, Los Angeles, CA USA; ^5^ Laboratory of Neuro Imaging, Stevens Neuroimaging and Informatics Institute, Keck School of Medicine, University of Southern California, Los Angeles, CA USA; ^6^ University of North Texas Health Science Center, Fort Worth, TX USA

## Abstract

**Background:**

Structural MRI can describe neurodegeneration associated with aging and Alzheimer Disease (AD). Brain age gap (BAG) quantifies the difference between chronological age and predicted “brain age” and can be estimated using many published algorithms. Higher BAG indicates accelerated brain aging. Regional estimates of volume and thickness detect structural differences in AD signature regions (e.g., hippocampus, tempo‐parietal regions). This project aimed to identify the structural MRI measure that had the best correspondence with Clinical Dementia Rating (CDR) in an ethno‐racially diverse sample.

**Method:**

We compared structural MRI measures in the Health & Aging Brain Study–Health Disparities (691 non‐Hispanic Black (NHB), 1094 Mexican‐American (MA) and 1085 non‐Hispanic White (NHW) participants). We calculated BAG using DeepBrainNet (BAG_DBN_) and BrainAgeR (BAG_BAR_). We derived Freesurfer‐based cortical thickness (meta‐ROI) and hippocampal volume (normalized for intracranial volume). We applied ANOVA followed by post‐hoc Tukey tests to assess differences in the interaction of self‐identified race/ethnicity and CDR, correcting for age, gender, and scanner. We performed ordinal regression to predict CDR using the same covariates, except CDR.

**Result:**

After correction for multiple comparisons, BAG_DBN_ significantly differed between CDR 0 and CDR 0.5 in MA (p_corrected_=0.045) and NHB (p_corrected_=0.017) but not NHW (p_corrected_=0.110). The other measures significantly differed between CDR 0 and CDR 0.5 for NHW only (Figure 1). For individuals with a 1 Z‐score elevation in BAG_DBN_ (6.1 years), NHW have 1.21 (95%CI: 1.18, 1.26) times the odds of being CDR 0.5, while NHB significantly greater risk (OR = 1.31, 95%CI: 1.27, 1.36). MA have 1.26 times the odds (95%CI: 1.22, 1.31). In contrast, for individuals with a 1 Z‐score decrease in cortical thickness, there is no difference in odds ratio by ethno‐racial group (NHW=1.21, 95%CI: 1.17, 1.27; NHB=1.30, 95%CI: 1.24, 1.37; MA=1.28, 95%CI: 1.24, 1.33) (Figure 2).

**Conclusion:**

The neurodegeneration measures with the strongest correspondence to CDR varied by ethno‐racial group. BAG_DBN_ may display greater sensitivity to the subtle changes that occur as individuals transition from CDR = 0 to 0.5 in underrepresented cohorts. Further work is needed to understand what features BAG_DBN_ captures, but this suggests care should be taken when choosing neurodegeneration markers.